# Design and Characterization of Functionalized Polyelectrolyte–Dicephalic Surfactant Complexes as Multipurpose Colloidal Systems

**DOI:** 10.1021/acsomega.5c12042

**Published:** 2026-02-13

**Authors:** Weronika Szczęsna-Górniak, Łukasz Lamch, Lucyna Hołysz, Piotr Warszyński, Kazimiera A. Wilk

**Affiliations:** † Department of Engineering and Technology of Chemical Processes, Faculty of Chemistry, 49567 Wrocław University of Science and Technology, Wrocław 50-370, Poland; ‡ Department of Physical Chemistry — Interfacial Phenomena, Faculty of Chemistry, 49686 Maria Curie-Sklodowska University, Lublin 20-031, Poland; § Jerzy Haber Institute of Catalysis and Surface Chemistry, 132074 Polish Academy of Sciences, Kraków 30-239, Poland

## Abstract

Polyelectrolyte–surfactant complexes (PESCs) have emerged as versatile soft-matter systems, offering unique opportunities for the design of multifunctional delivery platforms. Therefore, this study investigates the design, formation, and characterization of novel PESCs based on antimicrobial-functionalized poly­(acrylic acid) (PAA) derivatives and a newly synthesized cationic dicephalic surfactant, 2-dodecyl-*N,N,N,N’,N’,N’*-hexamethyl-propan-1,3-ammonium dibromide (C_12_-D_C_NMe_3_Br). Building on our previous work on antimicrobial-decorated PAAs grafted with thymol (PAA-THY-15), menthol (PAA-MEN-15), and carvacrol (PAA-CAR-15), these polyanions were combined with the oppositely charged surfactant to construct multipurpose carrier systems. The designed PESCs were loaded with curcumin (CUR), a model hydrophobic drug with therapeutic properties, to evaluate their potential applicability as drug delivery systems (DDSs). A variety of physicochemical techniques were applied to gain insight into the complexation processes, self-assembly behavior, and functional properties of the resulting PESCs. Surface activity of new complexes was assessed by goniometric measurements, while their colloidal stability over time was studied using the turbidimetric method. Dynamic light scattering (DLS) provided information on particle size, polydispersity, and surface charge. Encapsulation efficiency (EE) and release kinetics were assessed by UV–vis spectrophotometry to evaluate the ability of the complexes to effectively entrap CUR and provide sustained release. The integration of antibacterial PAA derivatives with dicephalic surfactants highlights the versatility of PESCs as tunable, multifunctional carriers that combine antimicrobial protection with controlled drug delivery. These findings demonstrate that the designed complexes are promising candidates for advanced DDSs and pave the way for further development of functional colloidal materials tailored for biomedical applications.

## Graphical abstract

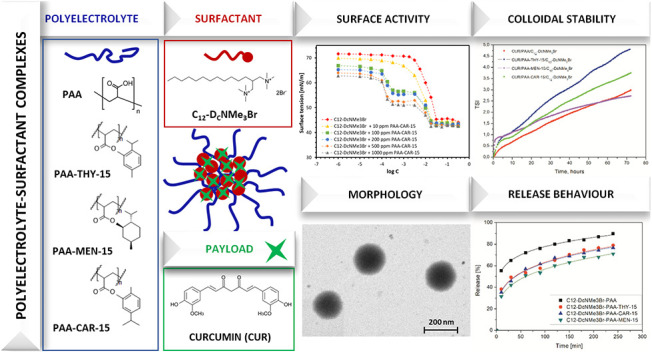

## 1 Introduction

In recent years, increasing attention has been focused on systems formed by the association of surfactants with polyelectrolytes (PEs) due to their unique self-assembly behavior and wide-ranging functional properties. Polyelectrolyte–surfactant complexes (PESCs) offer a versatile platform due to their remarkable diversity in structure, morphology, and physicochemical features. These attributes make them highly appealing for a variety of applications across different scientific and industrial fields, such as drug delivery, biotechnology, personal care, food formulation, environmental remediation, and advanced materials engineering.^1,2^

PESCs are colloidal assemblies formed through the self-organization of oppositely charged PEs and surfactants in aqueous media. Their formation results from the balance of attractive and repulsive forces. Key driving interactions include: (i) electrostatic attraction between the charged groups on the PE backbone and the ionic head groups of surfactant molecules; (ii) hydrophobic interactions among surfactant tails as well as between them and hydrophobic regions of the PE; (iii) entropy gain from the release of associated counterions during complex formation; (iv) suppression of repulsive forces between polar moieties within surfactant aggregates (micelles) upon binding to the PE; (v) reduced hydration of surfactant molecules due to their incorporation into polyelectrolyte–surfactant assemblies.^1,3^ The relative contribution of these interactions may affect the overall organization and interfacial characteristics of the complexes. Accordingly, electrokinetic parameters such as ζ potential should be considered indirect descriptors of colloidal behavior and may reflect differences in particle density, surface properties, or hydration rather than direct evidence of specific molecular arrangements. However, although PESC assembly is mainly driven by electrostatic attractions and hydrophobic forces, the overall association process is far more intricate, as it involves a delicate balance between multiple types of interactions such as steric effects, van der Waals interactions, and possibly hydrogen bonding.^4,5^

The structure and properties of PESCs are highly sensitive to a variety of parameters, including the molecular weight, flexibility and concentration of the PE, its charge density and degree of branching, the nature of the surfactant (e.g., tail length, headgroup), its concentration, the mixing ratio, as well as external conditions such as pH, ionic strength, and temperature.^4,5^ As a result, these complexes can adopt a range of mesoscopic morphologies – from simple spherical or rodlike micelle aggregates and coacervates to more complex lamellar bilayers or hierarchical structures with liquid-crystalline order.^1,4^

One of the defining features of PESCs is their hierarchical self-assembly behavior, which is often characterized by a transition from noncooperative to cooperative binding of surfactants as their concentration increases. Below the critical aggregation concentration (CAC), surfactant molecules tend to bind individually to the PE chains. Whereas above the CAC, micelle-like aggregates begin to form, often at concentrations significantly lower than the critical micelle concentration (CMC) of the pure surfactant.^3,5^ Formed PESCs contain small micellar domains that provide a hydrophobic environment suitable for the encapsulation and solubilization of poorly water-soluble active compounds. These complexes effectively combine the solubilizing capabilities of surfactants, which typically form nanoscale structures of 3–8 nm, with the larger dimensional characteristics of PEs, which can extend from approximately 20 nm up to several micrometres. The overall size of the resulting complexes generally correlates with the length of the PE chains, and the number of incorporated micellar aggregates can vary widely, from a few dozen to several thousand.^4^

Depending on the stoichiometry of the components and the solution conditions, PESCs can exist as soluble complexes, precipitates, or even form dispersed nanoparticles with core–shell architectures. Typically, the inner core consists of charge-compensated polyelectrolyte–surfactant aggregates, while the shell is composed of excess polymer chains or additional stabilizing agents.^4^ This versatile structural organization enables the fine-tuning of PESC properties for specific applications, such as drug delivery, where controlled release profiles and stability in physiological environments are critical.^1^ Moreover, combining surfactants with PEs in the form of PESCs results in larger aggregates with slower response dynamics, which is advantageous for controlled-release systems. This is particularly relevant in drug delivery applications, where pure surfactant systems may solubilize active substances effectively but tend to release them too rapidly, especially upon dilution, which typically decreases the surfactant concentration below the cmc. This not only enhances the stability and solubilization capacity of the complexes but also minimizes the concentration of free surfactant molecules in solution, thereby reducing potential toxicity.^4^

An additional and challenging aspect is understanding the interactions between PESCs and the drug intended for solubilization and delivery. Bioactive compounds exhibit a wide range of hydrophilicity and solubility profiles in both aqueous and hydrophobic media. One notable advantage of PESCs is their ability to provide a variety of solubilization sites not limited to those offered by surfactants or PEs. PESCs may encapsulate drug molecules that are both highly as well as poorly soluble in purely hydrophobic domains, such as the core of surfactant micelles, due to the heterogeneous internal PESCs structure, characterized by regions of different polarity and hydrophobicity. The effectiveness of these solubilization sites directly influences the binding affinity and incorporation efficiency of the active compound.^4^ Taken together, the above factors, including structural versatility, combined with tunable interactions and biocompatibility (depending on the choice of constituents) of PESC-based systems, make them promising candidates for drug delivery applications, particularly in designing tailor-made functional colloidal materials with controlled-release features.

The selection of the appropriate surfactant and PE is essential to design efficient PESC systems as pharmaceutical delivery platforms. Their chemical structure, charge, and ability to interact with drug molecules determine the effectiveness of encapsulation and release of active substance^6,4^ The strength and type of interaction between the surfactant and PE also affect the stability, size, and responsiveness of the system.^7^

The use of multicharged surfactants, particularly dicephalic surfactants, in the formation of PESCs offers several distinct advantages.^8^ Their multiple charged head groups enhance electrostatic interactions with oppositely charged PEs, leading to stronger complexation and generation of stable hydrophobic domains, which are particularly favorable for solubilization.^9^ Moreover, the structural complexity of dicephalic surfactants, characterized by two hydrophilic heads connected to a single hydrophobic tail, allows for improved control over the morphology and size of the resulting aggregates. This can result in more compact, organized, and functionally diverse assemblies.^2^ Such features are particularly advantageous in applications that require tailored release profiles, enhanced encapsulation efficiency, or increased stability of the complex under varying environmental conditions.

The functionalized PEs, especially those bearing hydrophobic moieties, offer significant advantages in the formation of PESC systems. Hydrophobically modified PEs introduce an additional driving force for self-assembly through hydrophobic interactions, which complements the electrostatic attraction between oppositely charged components. This dual interaction mechanism enhances the stability of the complexes and enables the formation of more compact and structured aggregates.^10^ Furthermore, the incorporation of hydrophobic segments provides new domains within the complex that can more easily entrap poorly water-soluble substances, thus improving the encapsulation and release profile of hydrophobic drug molecules.^1^ In addition, functionalized PEs can also play a protective antimicrobial role when modified with antibacterial groups. This feature is of particular significance for drug delivery systems intended for administration in infected or bacterial-contaminated environments, where microbial colonization may adversely affect therapeutic efficacy. Incorporation of antimicrobial moieties into PEs used in PESCs enables the development of multifunctional delivery systems that not only enhance drug solubilization and release but also inhibit bacterial growth, reducing the risk of secondary infections and improving treatment outcomes. The PEs decorated with antimicrobial functionality can provide sustained antibacterial activity through labile chemical linkages (e.g., hydrolyzable ester or amide bonds) that release active moieties under physiological conditions. This dual-function approach is particularly relevant in the context of rising antimicrobial resistance (AMR) and offers a promising strategy for enhancing the safety and efficacy of drug delivery platforms, while simultaneously impairing pathogen-induced complications. Examples of such functionalized PEs are the poly­(acrylic acid) (PAA) derivatives with antimicrobial function, including PAA grafted with thymol (THY), menthol (MEN), and carvacrol (CAR) that we have synthesized and thoroughly described in our recent publication.^11^ In that work, we presented their chemical structure, physicochemical and biological properties, as well as multifunctional potential of new PEs, particularly in the context of drug delivery systems and antimicrobial protection. Therefore, those PEs decorated with antibacterial function can be used as versatile building blocks for the development of advanced PESC-based delivery platforms.

In this paper, we aimed to study the complexation processes of curcumin (CUR)-loaded mixed systems comprising a cationic dicephalic surfactant and oppositely charged anionic PEs with antimicrobial activity, to form multipurpose DDSs. The compound consisting of a dodecyl alkyl chain and exclusively carbon atoms in its hydrophobic region, except for the hydrophilic quaternary ammonium head groups and associated counterions (C_12_-D_C_NMe_3_Br), was chosen as a novel class of dicephalic-type surfactant.^12^ PAA and its derivatives with antimicrobial function such as PAA decorated with essential oils including THY, MEN and CAR, (PAA-THY-15, PAA-MEN-15, PAA-CAR-15) were used as polyanions.^11^ These essential oil derivatives were selected due to their well-documented broad-spectrum antibacterial activity and low systemic toxicity compared to other antimicrobial agents, as well as their ability to interact hydrophobically with both cargo molecules and the polymer matrix, which can modulate drug release profiles and improve complex stability.^11^ CUR, a model hydrophobic compound with proven therapeutic potential, was selected for encapsulation within the complexes. A wide range of physicochemical techniques has been employed to obtain comprehensive insight into the properties of PESCs systems and understand their self-assembly mechanisms. Surface tension of novel PESCs was measured using a goniometric method to assess their interfacial activity. The resulting CUR-loaded PESCs were characterized in terms of their size and surface charge using dynamic light scattering (DLS) as well as colloidal stability over time using the turbidimetric method to provide an insight into their resistance to sedimentation, aggregation, and phase separation. Furthermore, encapsulation efficiency (EE) was quantified to evaluate the complexes’ ability to enclose the hydrophobic drug. Release studies were also performed to monitor the release kinetics of CUR over time, enabling assessment of the release capabilities of the developed delivery systems. The combination of the designed antibacterial PEs with oppositely charged dicephalic surfactants provides an attractive platform for exploring fundamental physicochemical interactions and developing tailor-made multifunctional colloidal materials applicable to a range of therapeutic areas, including antimicrobial and anticancer therapies.

## 2 Results and Discussion

### 2.1 Adsorption of Dicephalic Surfactant – C_12_-D_C_NMe_3_Br in the Presence of Anionic Polyelectrolytes with Antimicrobial Function

Understanding the formation mechanism of surfactant–polyelectrolyte complexes is crucial for the design of nanostructures driven by electrostatic interactions and plays a key role in the development of advanced colloidal systems. Therefore, surface tension measurements were conducted to evaluate the interfacial behavior of C_12_-DcNMe_3_Br, a cationic surfactant, in the presence of anionic polyelectrolytes such as PAA and its functionalized derivatives containing thymol (PAA-THY-15), menthol (PAA-MEN-15), and carvacrol (PAA-CAR-15). In each system analyzed, the dicephalic surfactant was combined with increasing concentrations (10–1000 ppm) of PAA or its hydrophobically modified derivatives. The structures of all used substances are shown in Scheme 1. The surface tension isotherms of studied complexes are presented in Figure 1.

**Scheme 1:**
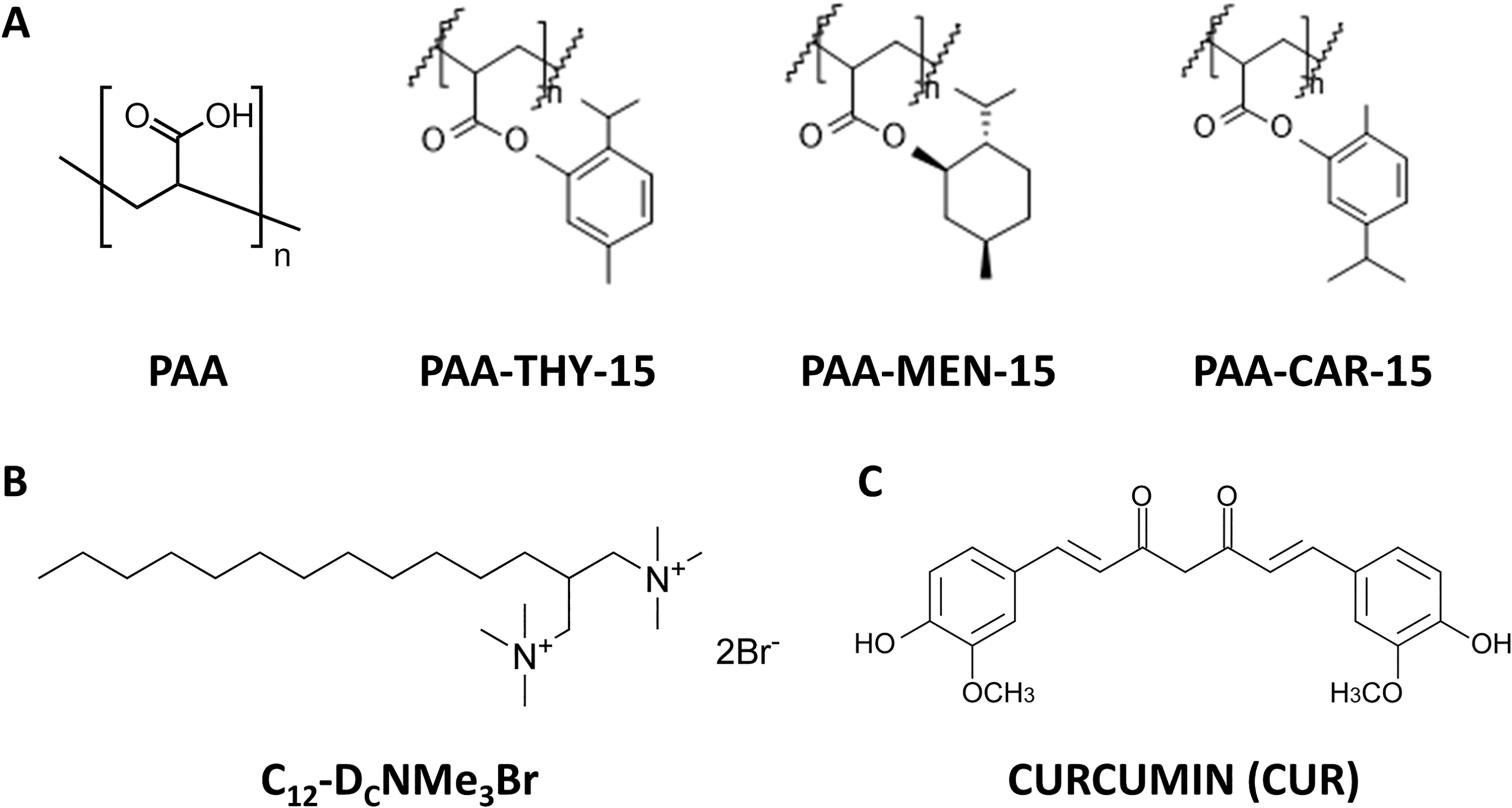
Structures of the Compounds Studied in Our Work: (A) Polyelectrolytes, (B) Surfactant, (C) Payload

**Figure 1:**
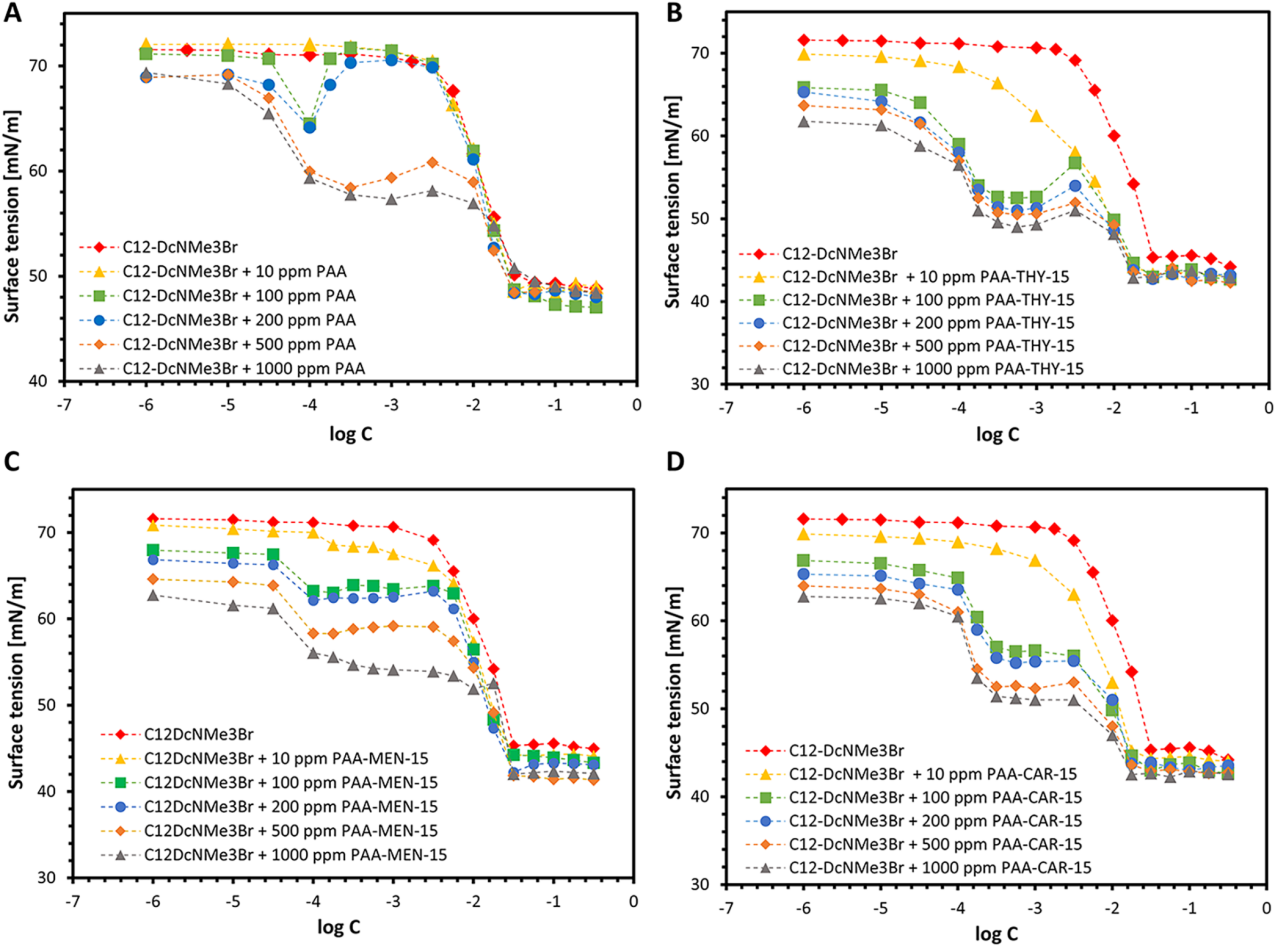
Surface tension isotherms of polyelectrolyte–surfactant mixed systems: (A) PAA/C_12_-D_C_NMe_3_Br; (B) PAA-THY-15/C_12_-D_C_NMe_3_Br; (C) PAA-MEN-15/C_12_-D_C_NMe_3_Br; (D) PAA-CAR-15/C_12_-D_C_NMe_3_Br.

The results revealed that functionalization and PE concentration influence surfactant–polyelectrolyte interactions and surface activity. In all cases, the addition of polyanions led to a notable reduction in surface tension compared to the surfactant alone, indicating strong interactions between the oppositely charged components and the formation of interfacial active complexes. The similar effect was observed and described in the literature.^13–15^

The first decrease in surface tension corresponds to the formation of polyelectrolyte–surfactant complexes in bulk solution, which are then adsorbed at the interface resulting in noticeable surface activity. The observed inflection point on the surface tension curve is referred to as the critical aggregation concentration (CAC) — the minimum surfactant concentration at which cooperative binding to the polyelectrolyte occurs, leading to the formation of PE-bound surfactant aggregates.^2,3^ The CAC indicates the threshold where electrostatic attraction between oppositely charged species transitions into organized complexation. For the given PE concentration, further increase of surfactant concentration does not induce a drop of the surface tension up to the concentration close to CMC — the concentration at which free micelles begin to form in solution. Then, some complexes at the interface are replaced by free surfactant, which is accompanied by a further decrease in the surface tension to the value corresponding to the surfactant CMC. At this stage, the PE becomes saturated with bound surfactant, and excess surfactant molecules begin to self-assemble into micelles independently of the PE.^2^

For all studied systems, the presence of increasing PE concentrations resulted in a progressive decrease in surface tension, reflecting enhanced adsorption of surfactant–polyelectrolyte aggregates at the air–water interface. The magnitude and position of the CAC varied depending on the chemical modification of the PAA (see Table 1). In the presence of unmodified PAA, the CAC is moderately reduced, reflecting typical electrostatic complexation (Figure 1A). A significantly more pronounced effect was observed in systems containing functionalized PEs. (Figure 1B–D).

**Table 1:** CMC and CAC Parameters of Polyelectrolyte–Surfactant Complexes

surfactant	polyelectrolyte	CAC [mM]	CMC [mM]
C_12_-DcNMe_3_Br	10 ppm PAA		31
	100 ppm PAA	0.10	32
	200 ppm PAA	0.10	29
	500 ppm PAA	0.11	29
	1000 ppm PAA	0.14	37
	10 ppm PAA-THY-15		19
	100 ppm PAA-THY-15	0.22	20
	200 ppm PAA-THY-15	0.23	20
	500 ppm PAA-THY-15	0.20	18
	1000 ppm PAA-THY-15	0.20	17
	10 ppm PAA-MEN-15	0.18	30
	100 ppm PAA-MEN-15	0.18	30
	200 ppm PAA-MEN-15	0.18	28
	500 ppm PAA-MEN-15	0.10	33
	1000 ppm PAA-MEN-15	0.13	31
	10 ppm PAA-CAR-15		19
	100 ppm PAA-CAR-15	0.24	20
	200 ppm PAA-CAR-15	0.25	18
	500 ppm PAA-CAR-15	0.18	19
	1000 ppm PAA-CAR-15	0.17	18

Functionalization of PAA with THY substantially enhanced the surface activity of the system. Compared to unmodified PAA, PAA–THY-15 induced a strong decrease in surface tension at lower surfactant concentrations, especially at 500 and 1000 ppm PAA–THY-15. This suggests that hydrophobic THY moieties enhance cooperative binding between the PE and the surfactant, probably through hydrophobic association, promoting earlier interfacial aggregation and micellization.^16,17^

The PAA–MEN-15 systems showed a similar trend, although the effect was less intense than in PAA-THY-15. The presence of MEN introduced a moderate hydrophobic character, enabling partial enhancement of polyelectrolyte–surfactant interaction. The surface tension began to decrease earlier than in the surfactant-only system, but the transition was broader, suggesting more gradual complex formation and weaker cooperative interactions compared to PAA–THY-15.

PAA functionalization with CAR produced a similar but slightly weaker effect than THY. That may suggest the role of molecular packing and steric effects in the formation of PESC.

Notably, in the systems containing functionalized PEs, the CMC appears at lower concentrations compared to the surfactant alone (CMC_C_12_‑DCNMe_3_Br_ = 31 mM) (see Table 1), indicating that some surface active PESC are still present at the interface.

These results confirm that both the presence and nature of hydrophobic moieties within the PE backbone play crucial role in modulating the interfacial properties of PESCs. The enhanced surface activity observed in the modified systems indicates their potential as efficient carriers for poorly water-soluble drugs and highlights their tunability through molecular design.

### 2.2 Colloidal Stability of the Functional Polyelectrolyte–Surfactant Complexes

PESCs have attracted increasing attention as carriers for poorly water-soluble active compounds due to their tunable physicochemical properties enabling improvement of the functional features; such as enhanced solubility, biocompatibility, low toxicity, as well as controlled drug release.^2,18^

The most important aspect of ensuring high efficiency of a medicinal product is its ability to maintain its original properties over time. Two parameters that jointly constitute the attribute of quality are necessary to determine these: durability and stability. From a functional perspective, assessing a drug’s durability involves evaluating its stability over a specific period, which allows determining the expiration date. This is particularly important in the case of colloidal systems, which are inherently thermodynamically unstable. When designing new colloidal drug delivery systems, special attention should be paid to the long-term stability of such systems, which is a critical property for practical application. Various methods are used to characterize the stability of colloidal systems. The most common are UV–vis spectroscopy, turbidimetry, dynamic light scattering, and density measurements.^19,20^

The colloidal stability can be considered regarding various aspects and mechanisms: kinetic, thermodynamic, electrostatic and steric (depletion). The “kinetic stability” is related to the existence of an energy barrier preventing coagulation, while the “thermodynamic stability” refers to the free energy difference between the coagulated and dispersed phases. They can be assessed, among other things, by examining changes in the hydrodynamic diameter, surface charge, or concentration of the colloidal particles over time, as well as an optical determination of possible destabilization processes taking place.

Further studies focused on the formation and characterization of functional polyelectrolyte–surfactant complexes loaded with CUR in order to assess their stability as advanced drug delivery systems. The physicochemical characterization of the studied PESCs presented in Table 2 highlights the influence of both CUR loading and PAA functionalization on the structural features of PESCs. The evaluated parameters, including mean diameter (MD), polydispersity index (PDI), and ζ potential (ZP) were measured directly after preparation and after 3 days of storage under ambient conditions.

**Table 2:** Characteristics of the Functional Polyelectrolyte–Surfactant Complexes^a^

PESC	MT [days]	MD [nm]	PDI	ZP [mV]	EE [%]
PAA/C_12_-DcNMe_3_Br	0	148.1 ± 2.3	0.264	–40.1 ± 1.8	
	3	191.1 ± 2.8	0.179	–38.7 ± 0.5	
CUR/PAA/C_12_-DcNMe_3_Br	0	165.4 ± 3.1	0.285	–42.2 ± 1.5	51.3 ± 2.5
	3	208.6 ± 4.0	0.168	–39.4 ± 0.9	
PAA-THY-15/C_12_-DcNMe_3_Br	0	150.0 ± 10.9	0.217	–67.5 ± 2.6	
	3	136.3 ± 0.9	0.163	–64.8 ± 1.8	
CUR/PAA-THY-15/C_12_-DcNMe_3_Br	0	161.2 ± 12.0	0.236	–65.2 ± 1.4	57.1 ± 3.1
	3	152.9 ± 2.1	0.178	–63.7 ± 1.3	
PAA-MEN-15/C_12_-DcNMe_3_Br	0	195.8 ± 1.0	0.183	–64.1 ± 2.3	
	3	191.6 ± 0.4	0.141	–62.6 ± 1.4	
CUR/PAA-MEN-15/C_12_-DcNMe_3_Br	0	216.5 ± 2.4	0.151	–61.2 ± 2.6	54.4 ± 1.8
	3	212.7 ± 1.2	0.127	–63.1 ± 3.5	
PAA-CAR-15/C_12_-DcNMe_3_Br	0	175.6 ± 1.2	0.123	–68.1 ± 2.2	
	3	172.9 ± 0.3	0.091	–66.3 ± 3.0	
CUR/PAA-CAR-15/C_12_-DcNMe_3_Br	0	192.8 ± 1.6	0.137	–69.5 ± 1.7	55.8 ± 2.2
	3	188.2 ± 0.9	0.103	–67.5 ± 2.7	

a MT – measurement time after preparation; MD – mean diameter; PDI – polydispersity index; ZP – ζ potential; EE – encapsulation efficiency.

The results collected in Table 2 show that functionalization of PEs with essential oil constituents (THY, CAR, MEN) induced some changes in colloidal structures, including greater particle sizes and increased ZP values.

The unloaded PESCs exhibited initial diameters in the range of 148–196 nm, depending on the type of functionalization. The initial particle sizes of loaded complexes were larger, ranging from 153 to 217 nm. This increase may be attributed to the additional hydrophobic interactions introduced by the essential oil moieties, potentially promoting looser or more swollen structures.^21^

The particle diameters of the unloaded and CUR-loaded complexes were comparable, in agreement with findings from other studies on related complexes.^20,22^ Upon storage (3 days), the majority of the formulations maintained their size, with only minor changes in MD values, indicating good colloidal stability of the systems. CUR/PAA/C_12_-D_C_NMe_3_Br showed a noticeable increase in particle size from 165.3 to 208.6 nm, which may reflect structural rearrangements or early aggregation tendencies. The curcumin-containing PESCs of functionalized PAA seem to shrink slightly, due to possible compaction of the complex, but that effect is close to the experimental error. Importantly, the PDI values remained low in all samples (≤0.285), confirming uniform size distributions and good colloidal homogeneity.

The shape features of the polyelectrolyte–surfactant complexes unloaded and loaded with CUR were examined using scanning transmission electron microscopy (STEM), and representative images are displayed in Figure 2. The results confirmed that all nanostructure displayed a spherical shape. The particle sizes observed in STEM were consistent with the MD values obtained from DLS measurements.

**Figure 2:**
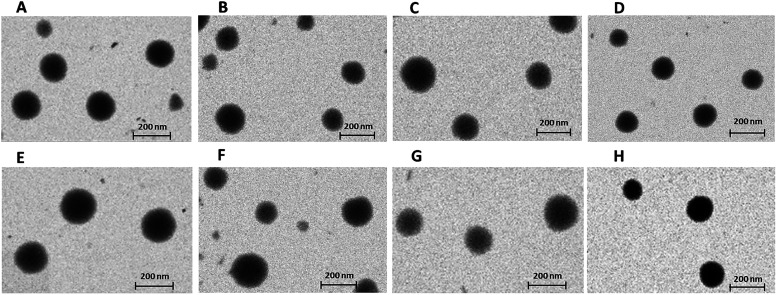
STEM images of polyelectrolyte–surfactant complexes: (A) CUR/PAA/C_12_-D_C_NMe_3_Br; (B) CUR/PAA-THY-15/C_12_-D_C_NMe_3_Br; (C) PAA/C_12_-D_C_NMe_3_Br; (D) PAA-THY-15/C_12_-D_C_NMe_3_Br; (E) CUR/PAA-MEN-15/C_12_-D_C_NMe_3_Br; (F) CUR/PAA-CAR-15/C_12_-D_C_NMe_3_Br; (G) PAA-MEN-15/C_12_-D_C_NMe_3_Br; (H) PAA-CAR-15/C_12_-D_C_NMe_3_Br.

In aqueous dispersions of nanoparticles their surface charge may determine the dispersion stability. However, if the nanosuspension is stabilized solely by the electrostatic repulsion, as s rule of thumb, a ζ potential of at least ± 30 mV of the particles is required for the suspension to be physically stable. In the case of combined electrostatic and steric stabilization, the value of ζ potential can be lower.^23^

The ζ potential values for all the systems were negative, indicating that the surface charge stabilization was primarily due to the polyanionic PAA backbone. The PAA/C_12_-D_C_NMe_3_Br complex displayed a moderately negative ζ potential (−38.7 mV), suggesting sufficient electrostatic repulsions to maintain the colloidal stability. The incorporation of CUR (CUR/PAA/C_12_-D_C_NMe_3_Br) did not change it. In contrast, the PESCs containing PAA functionalized with bioactive essential oil derivatives (THY, MEN, and CAR) exhibited markedly bigger negative ζ potential values, ranging from −61 to −69 mV. This significant increase in surface charge relative to that of the unmodified PAA-based complex indicates that the presence of hydrophobic moieties changes the PESCs structure with the exposure of PE free charged groups to the aqueous environment. The most negative potential was observed for the PAA-THY-15/C_12_-DcNMe_3_Br and PAA-CAR-15/C_12_-DcNMe_3_Br (unloaded and loaded with CUR), which can be associated with the specific physicochemical interactions between THY or CAR moieties of the PE matrix, potentially increasing the degree of ionization and exposing negatively charged functional groups on the PESC surface. Such high negative ζ potential values indicate an enhanced colloidal stability of the suspensions, resulting from the repulsive interactions. This is favorable in terms of their storage and biological applications. Similar conclusions were deduced by researchers investigating nanoparticles of cetylpyridinium chloride–alginate complex loaded with ibuprofen.^18^

Loading CUR into the modified systems caused only a minor shift in the ζ potential values (typically 1–3 mV), suggesting that CUR encapsulation does not significantly alter the surface charge of the complexes. Importantly, the ζ potential values remained relatively stable after 3 days of storage, with only a minor decrease of their negative values (2–3 mV on average). This observation indicates that no substantial aggregation or charge neutralization occurred over time, confirming the colloidal stability of both unloaded and CUR-loaded complexes for a long time. The encapsulation efficiency (EE) exceeded 51% in all studied systems, highlighting affinity of CUR with the polyelectrolyte–surfactant matrix. However, CUR encapsulation was more efficient in PESCs containing PAA functionalized with THY, MEN, and CAR than unmodified PAA, probably because the additional hydrophobic domains provide favorable sites for CUR entrapment and enhance drug–carrier compatibility.^11^

Dispersion stability experiments of the designed PESCs with CUR were conducted applying the turbimetric method with using the Turbiscan Lab Expert apparatus, which employs the multiple light scattering (MLS) phenomenon. This enabled analysis of the sample aging by recording the changes and instabilities via its flocculation and sedimentation. Measurements of the intensity of the light beam transmitted and scattered by the sample during scanning allowed detection of changes in the particle size at different heights in the analyzed sample.

The time-dependent transmission changes for the designed PESCs with CUR are provided in the Supporting Information (SI) (Figure S2), while Figure 3 shows changes in Turbiscan Stability Index (TSI) determined from eq 1 based on scattered light profiles. The TSI parameter is a quantitative measure of formulation stability, with low values generally corresponding to high physical stability and minimal destabilization phenomena.^19,24^

**Figure 3:**
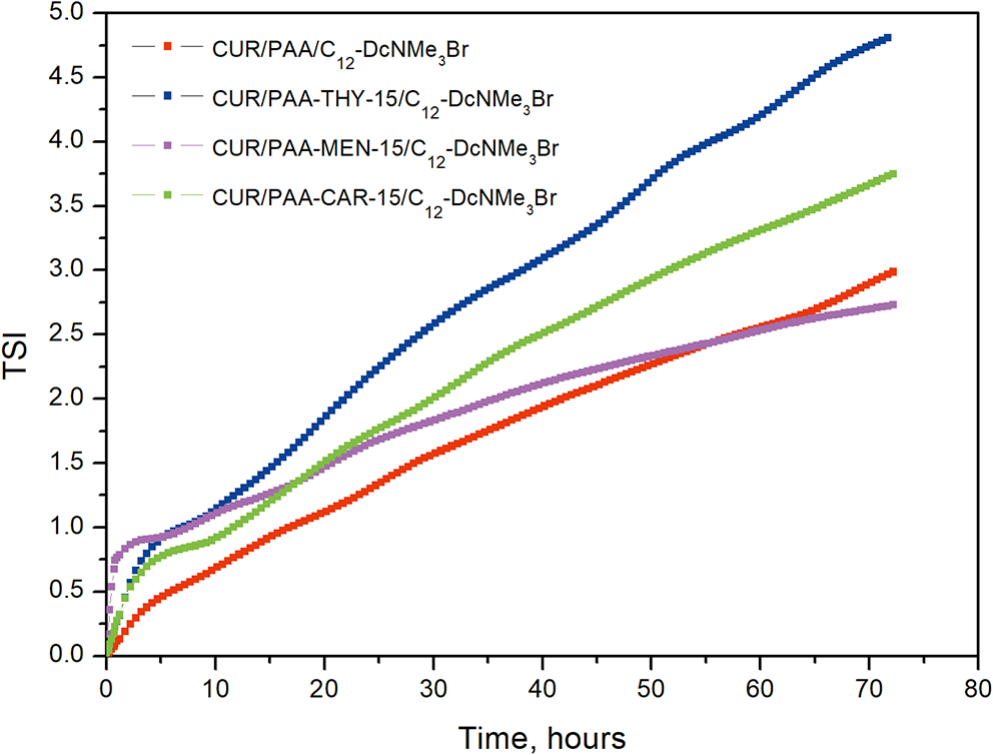
Changes in TSI over time for the studied polyelectrolyte–surfactant complexes.

Lower TSI values indicate fewer time-dependent changes in the spatial distribution of backscattering intensity and thus higher resistance to physical destabilization processes such as migration or phase separation. Therefore, TSI was used as a comparative indicator of physical aging among formulations measured under identical conditions. It should be noted that DLS probes the average hydrodynamic size of dispersed particles after dilution, whereas Turbiscan detects time-dependent spatial redistribution of scattering intensity in the undiluted sample; therefore, changes in TSI do not necessarily imply aggregation or size growth detectable by DLS.

The CUR/PAA/C_12_-D_C_NMe_3_Br complex demonstrated the most favorable stability profile, with TSI values gradually increasing to 3 within 72 h. That is despite the lowest ζ potential value. The addition of essential oils (MEN, THY, and CAR) to PAA resulted in a slight deterioration in the stability of PESCs with curcumin. During the first 12 h, TSI changes were similar, but with aging, destabilization processes began slowly, and this parameter increased almost linearly, faster for CUR/PAA-THY-15/C_12_-D_C_NMe_3_Br than for CUR/PAA-CAR-15/C_12_-D_C_NMe_3_Br. The CUR/PAA-MEN-15/C_12_-D_C_NMe_3_Br systems appeared to be the most stable. The CUR/PAA-THY-15/C_12_-D_C_NMe_3_Br complex, on the other hand, exhibited the least desirable stability profile, with an increase in TSI to 4.75 within 72 h. This trend reflects a slight loss of stability, likely due to structural rearrangements or separation processes induced by the presence of THY.

Summarizing, the above results revealed a clear structure–property relationship. That is, the nature of the hydrophobic substituent grafted onto the PAA backbone dictates the balance between colloidal stability, particle size, and drug encapsulation. The choice of hydrophobic moiety plays a crucial role in tailoring the physicochemical properties and shelf life of PESCs.

Among the studied systems, the complexes containing PAA functionalized with MEN (CUR/PAA-MEN-15/C_12_-DcNMe_3_Br) and CAR (CUR/PAA-CAR-15/C_12_-DcNMe_3_Br) exhibited superior colloidal stability compared to their THY-modified counterparts. This was evidenced by their more consistent and stable ZP values, lower standard deviations, and improved colloidal stability over time. From a drug delivery perspective, MEN- and CAR-functionalized systems may therefore represent promising nanocarriers of hydrophobic active component, combining colloidal stability with efficient drug loading.

### 2.3 Curcumin Release Behavior

The mechanism of payload release under specific conditions is crucial for assessing the ability of the polyelectrolyte–surfactant complexes to deliver the active compound.^25^ The release pattern of the encapsulated substance is influenced by multiple factors, including the nature of the payload, carrier properties, and formation processing parameters.^26^ The release behavior of designed PESCs was examined in PBS at pH 7.4 to assess their potential as carriers. The CUR release profiles of the studied PESCs are presented in Figure 4.

**Figure 4:**
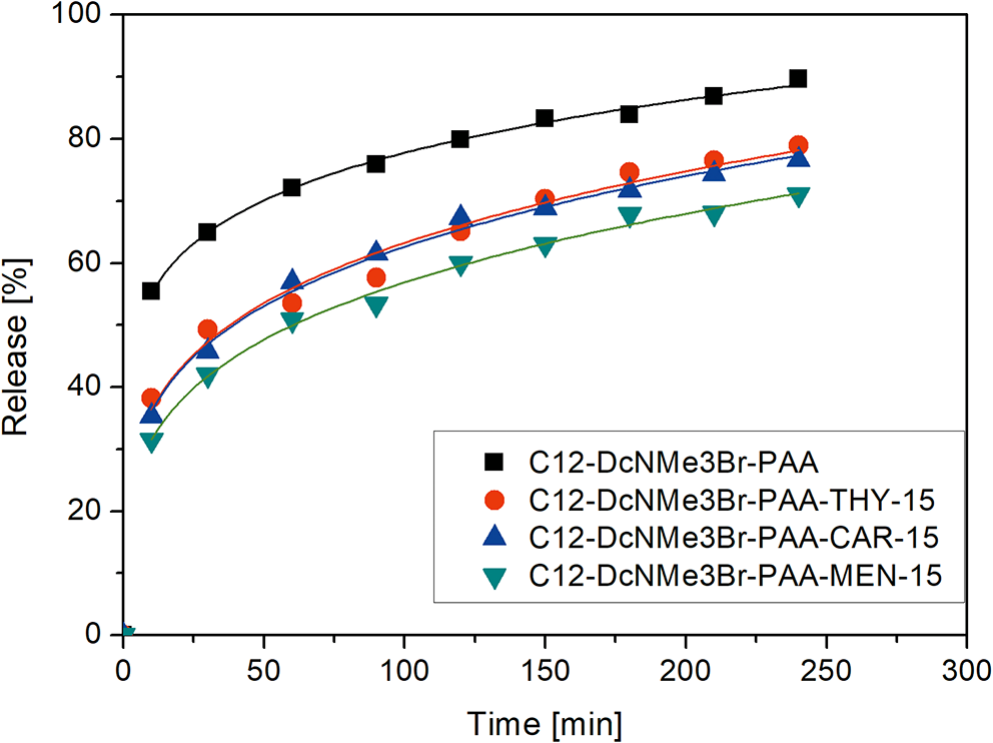
Release profiles of CUR from polyelectrolyte–surfactant complexes in PBS at 37 °C.

The C_12_-DcNMe_3_Br–PAA complex exhibits the fastest release, reaching approximately 85% after 300 min, which results from relatively weak interactions between CUR molecules and the polyelectrolyte–surfactant matrix. In contrast, the complexes containing PEs functionalized with the essential oil constituents (THY, CAR, MEN) reduced the overall release degree to about 70–75%, suggesting a stabilizing effect arising from the hydrophobic functional moieties. The presence of THY, CAR, and MEN enhances hydrophobic and π–π interactions within the complex, leading to stronger molecular packing and consequently restricting CUR diffusion into the aqueous phase.

The release profiles exhibit a biphasic character with an initial burst effect followed by a slower diffusion-controlled phase that dominates over longer time scales. The first mechanism can be attributed to the desorption of CUR weakly associated with the surface of the complexes. The complexes based on essential oil-modified PEs demonstrated a visibly slower release profiles (t_0.5_ = 33–56 min) compared to the PAA-based system (t_0.5_ < 10 min). The observed delayed release in functionalized PESCs suggests that structural modifications can be used to tailor drug release kinetics to therapeutic requirements.

The release data were analyzed using the Korsmeyer–Peppas (K–P) model, which is commonly applied to describe drug release kinetics from polymeric systems.^25^ The K–P model uses the release rate constant (k) to describe the overall speed of release and the release exponent coefficient (n) to characterize the mechanism of drug release from a carrier.^11,27^ The fitting parameters shown in Table 3 demonstrate distinct differences between the functionalized and nonfunctionalized complexes. The high values of the calculated correlation coefficient (*R* ^2^ = 0.974–0.996) confirm an excellent fit of the K–P model to the experimental data. The PAA-based system exhibits the highest kinetic constant (*k*_m_ = 39.01), reflecting fast CUR diffusion through the less compact matrix. In contrast, the complexes containing PEs functionalized with THY, CAR, or MEN display lower kinetic constants (*k*_m_ = 17.47–20.84), confirming a slower release process.

**Table 3:** Kinetic Parameters Obtained by Fitting the Korsmeyer-Peppas Model to the CUR Release Data from Functionalized Complexes

complex				Korsmeyer–Peppas parameters		
payload	surfactant	polyelectrolyte	t_0.5_ [min]	*k*_m_ [min^–n^]	*n*	adj. *R* ^2^
CUR	C_12_-DcNMe_3_Br	PAA	<10	39.01 ± 0.63	0.15 ± 0.01	0.996
		PAA-THY-15	33	20.84 ± 1.59	0.24 ± 0.02	0.974
		PAA-MEN-15	56	17.47 ± 0.76	0.26 ± 0.01	0.992
		PAA-CAR-15	42	20.74 ± 0.80	0.24 ± 0.01	0.993

The release exponent coefficient (*n*) ranges from 0.15 to 0.26 for all studied systems, indicating Fickian diffusion-controlled release.^11,28^ This demonstrates that CUR transport that occurs through the polyelectrolyte–surfactant complexes is driven by concentration gradients, while the presence of essential oil constituents primarily alters the compactness and hydrophobicity of the complexes.

These findings indicate that functionalization of the polyelectrolyte with essential oil derivatives leads to a reduced initial burst release of CUR from PESCs, likely due to additional hydrophobic interactions, while the subsequent release follows a similar diffusion-driven profile for both modified and nonfunctionalized complexes. Overall, the above analysis highlights the potential of functional PESCs containing bioactive hydrophobic moieties as promising carriers for hydrophobic drugs such as CUR. The tunable release behavior, governed by molecular interactions within the complexes, enables design delivery systems with controlled and sustained drug release.

## 3 Conclusions

This study reports the successful development and comprehensive characterization of CUR-loaded polyelectrolyte–surfactant complexes (PESCs) constructed from antimicrobial-functionalized poly­(acrylic acid) (PAA) derivatives and a dicephalic surfactant. The anionic components were represented by PAAs grafted with thymol (PAA-THY-15), menthol (PAA-MEN-15), and carvacrol (PAA-CAR-15), while the cationic counterpart was the surfactant C_12_-DcNMe_3_Br. The findings confirm that both the presence and the structural identity of hydrophobic substituents within the PE backbone are critical in shaping the interfacial properties of PESCs. The enhanced surface activity of functional complexes achieved through hydrophobic modifications underlines their potential as efficient drug delivery systems. The results clearly demonstrate that the chemical nature of the antimicrobial moieties of decorated PAA profoundly affects the physicochemical behavior and overall performance of the obtained PESCs. Notably, complexes containing MEN- and CAR-modified PAAs displayed superior colloidal stability compared with THY-based systems, as supported by turbidimetric analysis. It indicates that suitable functionalization strategies can effectively reduce structural heterogeneity and ensure greater durability of the systems under aqueous conditions. All complexes based on PEs grafted with essential oil-derived moieties demonstrated moderately high encapsulation efficiency and sustained, diffusion-driven release profiles, outperforming the unmodified PAA-based systems. The presence of hydrophobic groups within the PE backbone not only improved drug loading but also ensured a more controlled release of CUR, underscoring the importance of hydrophobic interactions in regulating release kinetics. It was confirmed that functionalized PESCs exhibit favorable performance characteristics and can be suitable candidates for effective carriers for poorly water-soluble drugs, with tunable release properties.

In conclusion, incorporating bioactive functional groups into the polyelectrolyte, along with a dicephalic surfactant, provides a versatile approach for developing advanced drug delivery platforms. The combined benefits of improved colloidal stability and enhanced drug release behavior make the novel PESCs promising candidates for next-generation multifunctional carriers, capable of delivering antimicrobial protection alongside effective therapeutic delivery.

## 4 Materials and Experimental

### 4.1 Materials

The dicephalic 2-dodecyl-*N,N,N,N’,N’,N’*-hexamethyl-propan-1,3-ammonium dibromide (C_12_-D_C_NMe_3_Br) was synthesized as described and presented in Scheme S1 in SI, while its characterization by ^1^H NMR and FT-IR is shown in Figure S1 (SI). The details of the surfactant synthesis and characterization were described in our previous study.^12^ The synthetic routes for poly­(acrylic acid) (PAA) grafted with thymol (THY), menthol (MEN), and carvacrol (CAR) (PAA-X-15 (X = THY, MEN, CAR)) were shown in Scheme S2 in SI. The synthesis and characterization of PAA-X-15 were described in detail in.^11^

Thymol (purity >98,5%) (THY) and menthol (MEN) were purchased from Sigma-Aldrich (Poznań, Poland). Poly­(acrylic acid) (Mw = 100 kDa) (PAA) and carvacrol (CAR) were obtained from Pol-Aura (Zabrze, Polska). *N*,*N*’-dicyclohexylcarbodiimide (DCC) and 4-dimethylaminopyridine (DMAP) were synthetic grade and purchased from Acros Organics (Geel, Belgium). Curcumin (CUR) was obtained from Archem Sp. z o.o. (Kamieniec Wrocławski, Poland). Solvents and inorganic salts were obtained from Avantor Performance Materials (Gliwice, Poland).

### 4.2 Preparation of Polyelectrolyte–Surfactant Complexes

Four series of PESCs were prepared. Aqueous stock solutions of the surfactant (C_12_-D_C_NMe_3_Br) and the polyelectrolytes (PAA, PAA-THY-15, PAA-MEN-15, PAA-CAR-15) were prepared by weighing the proper amounts of dry substances. For each sample, polyelectrolyte and surfactant solutions were prepared at twice the concentration needed for the final mixture. Equal volumes of those solutions were mixed by the slow addition of polyelectrolytes to the surfactant solution. The final concentration of each compound was half that of the stock solution applied. The dispersions were stirred under magnetic stirring for 30 min, and then kept for 24 h to equilibrate.

### 4.3 Solubilization of CUR in Polyelectrolyte–Surfactant Complexes

CUR-containing complexes were obtained by adding 50 mg of CUR to the surfactant solutions, followed by their mixing with the selected polyelectrolyte solution, in the same way as empty polyelectrolyte–surfactant complexes were prepared. The surfactant concentration was selected equal to twice the CAC, while the polyelectrolytes’ concentration was constant at 1000 ppm. The surfactant-to-polyelectrolyte ratio (S/P) was 0.036, 0.021, 0.032, and 0.024 for PAA, PAA-THY-15, PAA-MEN-15, and PAA-CAR-15, respectively. The systems were stirred under magnetic stirring for 30 min, and then left for 24 h to equilibrate. The nonsolubilized fraction was removed from solutions by centrifugation (10 000 rpm, 10 min).

### 4.4 Characterization of Polyelectrolyte–Surfactant Complexes

#### 4.4.1 Surface Tension Measurements

The surface tension of polyelectrolyte–surfactant systems was measured at 295 K using the pendant drop shape analysis method based on fitting to Young–Laplace equation. The measurements were performed using DSA25 Expert Goniometer (Krüss, Germany), equipped with humidity control, Peltier-controlled temperature chamber, and ADVANCE Software for data analysis. The polyelectrolyte–surfactant solutions were prepared 24 h before measurements. Surface tension values were calculated as averages of at least 10 measurements.

#### 4.4.2 Mean Diameter, Polydispersity Index, ζ Potential and Morphology

The mean diameter (MD), polydispersity (PDI), and ζ potential (ZP) of the prepared PESCs were determined by dynamic light scattering (DLS) using the Zetasizer Nano ZS (Malvern Instruments, UK). The ζ potential was calculated from the electrophoretic mobility using the Smoluchowski approximation.^29^ Before DLS measurements, all samples were diluted with distilled water. The measurements were performed in triplicate at 22 °C, and the data are presented as means.

The morphology of the studied PESCs was visualized using a scanning transmission electron microscopy (STEM) (Thermo Scientific Scios 2 DualBeam system) operated at an accelerating voltage of 10 kV and a working distance of 8.0 mm. Bright-field STEM images were acquired using the STEM 3 detector at a beam current of 50 pA. Prior to analysis, the prepared samples were diluted in double-distilled water to a concentration of 0.1% w/w, deposited onto a clean glass surface, and allowed to adsorb for several minutes. Subsequently, the samples were air-dried at room temperature in a dust-free environment.

#### 4.4.3 Turbidimetric Measurements

The physical stability of studied PESCs was measured using a Turbiscan Lab Expert analyzer equipped with a temperature-controlled Turbiscan LAB Cooler module (Formulation, Toulouse, France). The dispersion sample (10 cm^3^) in a cylindrical glass vial was placed in the chamber of the Turbiscan Lab device and scanned for 72 h (every 10 min for the first hour and then every 30 min).

The principle of the Turbiscan measurement is based on the turbidimetric method. The laser light (880 nm) passing through the studied system is absorbed and/or reflected, and the signal is collected by two synchronized detectors that record the transmitted (T) and backscattered (BS) light signals as a function of time. This allows for rapid and sensitive identification of the destabilization mechanisms under controlled temperature conditions during the aging process. Based on the variation of the backscattering intensity in subsequent scans, the dimensionless parameter of the Turbiscan Stability Index (TSI) was determined according to the formula below, using a special computer program Turbiscan Easy Soft 2.3.

$$\mathrm{TSI}=\sqrt{\frac{\sum_{i=1}^{n}{\left(x_{i-x_{\mathrm{BS}}}\right)}^{2}}{n-1}}$$

where *x*_*i*_ is the average backscattering for each successive reading of the measurement, *x*_BS_ is the average *x*_*i*_ and *n* is the number of scans (repetitions of single measurement during the total time of the experiment).

TSI values may change from 0 (highly stable system) to 100 (extremely unstable system). According to the manufacturer’s guidelines, TSI values above 3 indicate visually detectable instability.^19^

#### 4.4.4 Encapsulation Efficiency

The encapsulation efficiency (EE) of CUR incorporated in PESCs was determined by UV–vis spectroscopy using a Hitachi U-2900 spectrophotometer. Absorption spectra were recorded in the 200–1000 nm wavelength range at a scanning speed of 800 nm/min. After the centrifugation of prepared PESCs, the supernatant was properly diluted using acetone, and the amount of CUR was determined spectrophotometrically at the wavelength of 419 nm using a previously prepared calibration curve. Measurements were performed in triplicate at 25 °C. The encapsulation efficiency was calculated using the following equation

$$\mathrm{EE}=\frac{m_{i}-m_{s}}{m_{i}}$$

where *m*_S_ is the mass of CUR present in the supernatant, and *m*_i_ is the initial mass of CUR used for the preparation of PESCs.

#### 4.4.5 Curcumin Release Study

The release of CUR from PESCs was studied in phosphate buffer saline (PBS) solution (pH 7.4) at 37 °C. Initially, the CUR-loaded nanostructures were suspended in PBS, transferred into a dialysis bag (MWCO = 3500 Da), and submerged in a glass vial filled with buffer solution. The complexes were incubated at 37 °C and stirred at 200 rpm. Samples (0.3 mL) were taken from the medium at predetermined time points, followed by replacement with an equal volume of PBS. The amount of released CUR was calculated based on the measurements of absorbance at λ = 419 nm using a UV–vis spectrophotometer (Hitachi U-2900). Experimental procedures were performed twice. The data were analyzed using the Korsmeyer–Peppas (KP) model.^25^

## Supplementary Material



## Notes

The synthetic routes for the dicephalic surfactant (C_12_-D_C_NMe_3_Br) (Scheme S1); FT-IR (up) and ^1^H NMR (down) spectra for the dicephalic surfactant (C_12_-D_C_NMe_3_Br) (Figure S1); synthesis of poly­(acrylic acid) functionalized with (a) thymol, (b) menthol, and (c) carvacrol obtained by Steglich esterification (Scheme S2); Turbiscan transmission profiles of the designed PESCs with curcumin (A) CUR/PAA/C_12_-D_C_NMe_3_Br, (B) CUR/PAA-THY-15/C_12_-D_C_NMe_3_Br, (C) CUR/PAA-MEN-15/C_12_-D_C_NMe_3_Br and (D) CUR/PAA-CAR-15/C_12_-D_C_NMe_3_Br (Figure S2) (PDF)
