# Supplementary material for: Design and Characterization of Functionalized Polyelectrolyte–Dicephalic Surfactant Complexes as Multipurpose Colloidal Systems
Source: ACS Omega. 2026 Feb 13;11(7):12415–25. doi: 10.1021/acsomega.5c12042 (PMC12947039; doi:10.1021/acsomega.5c12042)
Supplement: Supplementary file 1 [file ao5c12042_si_001.pdf]

Electronic Supplementary Material (ESI)

Design and Characterization of Functionalized Polyelectrolyte – Dicephalic Surfactant Complexes as Multipurpose Colloidal Systems

Weronika Szczęsna-Górniak[a], Łukasz Lamch[a], Lucyna Hołysz[b], Piotr Warszyński[c], Kazimiera A. Wilk[a]

[a] Department of Engineering and Technology of Chemical Processes, Faculty of Chemistry, Wrocław University of Science and Technology, Wrocław 50-370, Poland
[b] Department of Physical Chemistry — Interfacial Phenomena, Faculty of Chemistry, Maria Curie-Sklodowska University, Lublin 20-031, Poland
[c] Jerzy Haber Institute of Catalysis and Surface Chemistry, Polish Academy of Sciences, Kraków 30-239, Poland

Table of Contents

Scheme S1. The synthetic routes for the dicephalic surfactant (C_{12}-D_CNMe_3Br).

Figure S1. FT-IR (up) and ^{1}H NMR (down) spectra for the dicephalic surfactant (C_{12}-D_CNMe_3Br).

Scheme S2. Synthesis of poly(acrylic acid) functionalised with a) thymol, b) mentol, and c) carvacrol obtained by Steglich esterification.

Figure S2. Turbiscan transmission profiles of the designed PESCs with curcumin A) CUR/PAA/C_{12}-D_CNMe_3Br; B) CUR/PAA-THY-15/C_{12}-D_CNMe_3Br, C) CUR/PAA-MEN-15/C_{12}-D_CNMe_3Br and D) CUR/PAA-CAR-15/C_{12}-D_CNMe_3Br.

The synthesis of 2-dodecyl-N,N,N,N',N',N'-hexamethylpropan-1,3-ammonium dibromides was carried out in four steps: (i) alkylation of dimethyl malonate, (ii) reduction of the intermediate, (iii) substitution of hydroxyl groups with bromides, and (iv) final quaternization with trimethylamine (see Scheme S1). In the alkylation step, the reaction was performed in methanol (molar ratio of bromododecane:dimethyl malonate:sodium was 1:1.05:1.05) After refluxing for 8 h, the product (dimethyl 2-dodecylmalonate) was obtained by liquid–liquid extraction (water/diethyl ether) with 97% yield. This intermediate was subsequently reduced to the corresponding 2-dodecylpropane-1,3-diol using lithium aluminum hydride (50% molar excess) in tetrahydrofuran – yield: 83%. The resulting diol was further purified by recrystallization from hexane. Hydroxyl-to-bromide substitution was performed under solvent-free conditions (heating at 130–140 °C for 24 h) with PBr_3 in a 1:1 molar ratio to the substrate. The resulting 1-bromo-2-(bromomethyl)tetradecane was isolated by liquid–liquid extraction (water/diethyl ether) with yield of 91%. In the final step, quaternization, 1-bromo-2-(bromomethyl)tetradecane was reacted with a large excess of trimethylamine (430 mL of 30% solution in absolute ethanol mixed with 430 mL of ethyl acetate per 1 mol dibromide derivative) in a pressure vessel. After heating at 75–85 °C for 36 h, the mixture was kept in a refrigerator, and the precipitate—2-dodecyl-N,N,N,N',N',N'-hexamethylpropan-1,3-ammonium dibromide—was collected in 79.1% yield. For the synthetic route see Scheme S1. The characterization of the final product (C_{12}-$D_C NMe_3Br$) was performed by means of ^1H NMR (spectra for 5 mg/mL solutions in DMSO-d_6 were recorded on Bruker AMX-500 spectrometer, chemical shifts given in ppm are referred to TMS as an internal standard) and FT-IR (spectra for pellets in KBr were recorded on Bruker Vertex 70 instrument with bandwidth 4000 – 400 cm^{-1}) techniques – see Figure S1 for the results.

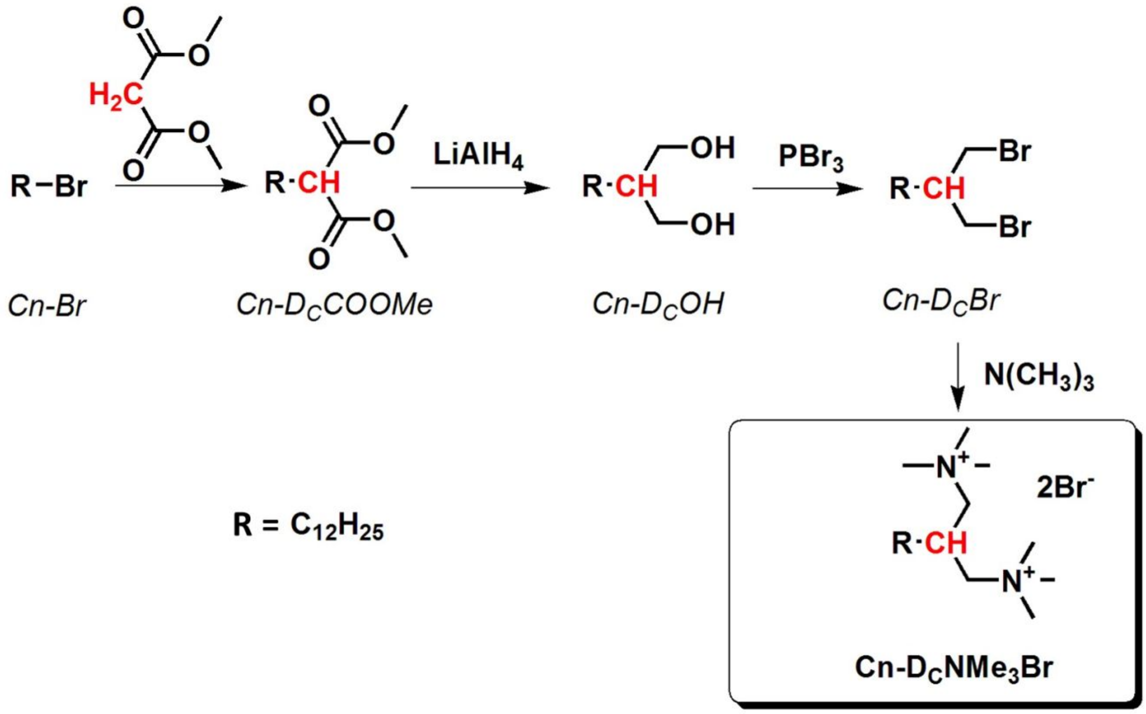

Scheme S1. *The synthetic routes for the dicephalic surfactant (C_{12}-$D_C NMe_3Br$).*

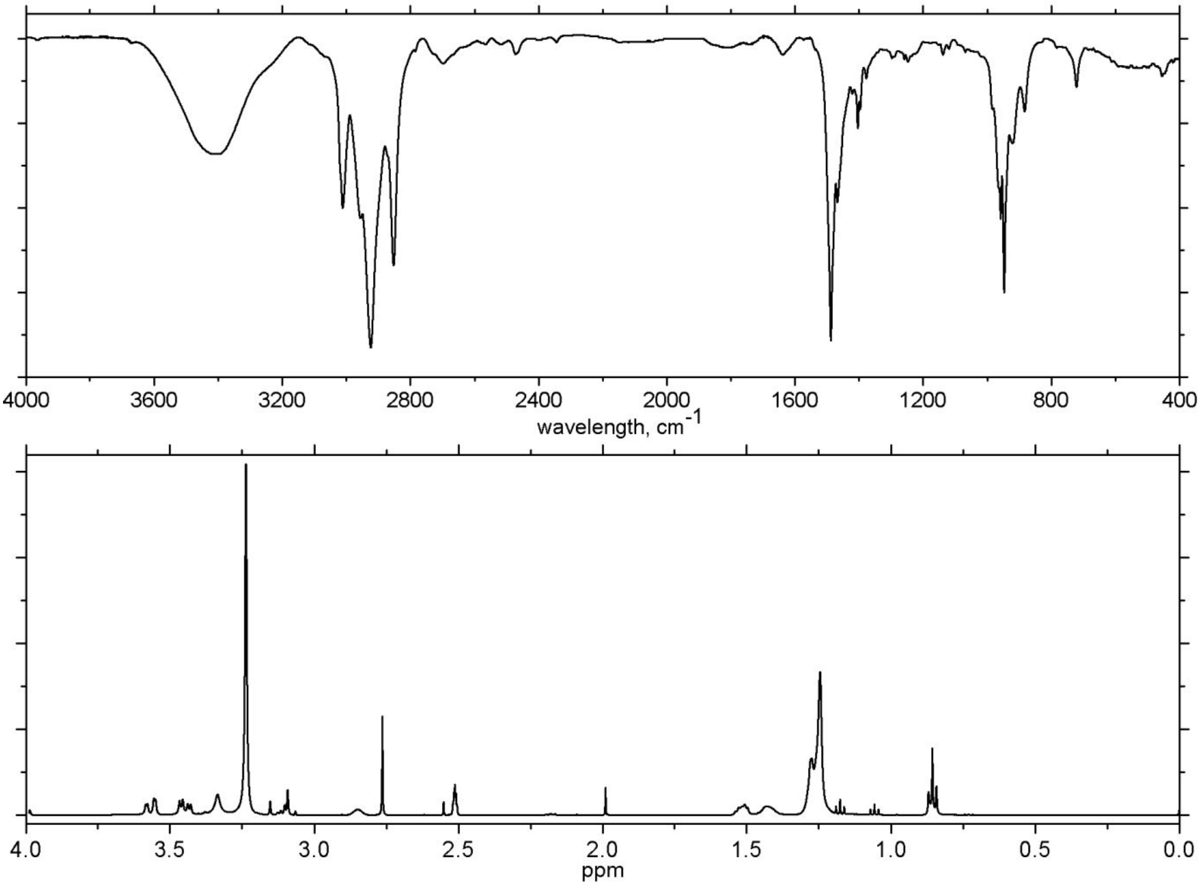

Figure S1. *FT-IR (up) and ^{1}H NMR (down) spectra for the dicephalic surfactant (C_{12}-D_CNMe_3Br).*

In ^{1}H NMR spectrum for C_{12}-D_CNMe_3Br four groups of signal are visible: triplet at 0.85 ppm, attributed to methyl group at the end of dodecyl chain, multiplets at 1.2 – 1.5 ppm (methylene motifs within dodecyl chain), multiplet at 2.75 ppm (methine group) as well as overlapping peaks at 3.2 ppm and 3.4 – 3.6 ppm (methyl and methylene groups neighboring with quaternary ammonium nitrogen within hydrophilic headgroups). Additional signals at ca 2.5 ppm and 2.0 ppm are attributed to deuterated solvent residual peak and acetone (used for NMR tubes cleaning), respectively. In FT-IR spectra there can be seen sharp signals at 2850–3015 cm^{-1} and 1400–1490 cm^{-1}, corresponding with C-H stretching and C-H bending / scissoring vibrations. The spectra confirmed the structure of C_{12}-D_CNMe_3Br as well as its purity (lack of any additional signals in the spectra, with an exception for solvent residual peaks in ^{1}H NMR).

Poly(acrylic acid) (PAA) modified with thymol (THY), menthol (MEN), or carvacrol (CAR) was obtained via Steglich esterification under mild conditions to prepare polyelectrolytes functionalized with antimicrobial groups (see Scheme S2). In brief: 41.7 mmol of carboxylic acid groups from PAA and 29.2 mmol THY, MEN or CAR were dissolved in 100–200 mL of DMSO, along with the required amount of 37.7 mmol N,N'-dicyclohexylcarbodiimide (DCC) and a catalytic quantity of 4-dimethylaminopyridine (DMAP). The molar ratio of PAA carboxylic acid groups to essential oil (THY, MEN, or CAR) to DCC was 1:0.7:0.9. The mixture was stirred at 22 °C for 48 h. To decompose unreacted DCC, 2 mL of distilled water was then added dropwise, followed by an additional 2

h of stirring. The precipitated by-product, dicyclohexylurea (DCU), was removed by filtration. The filtrate was dialyzed against distilled water (4 × 5 L, 3 days, MWCO = 3500 Da). Finally, the resulting solution was filtered, and the product was recovered by lyophilisation.

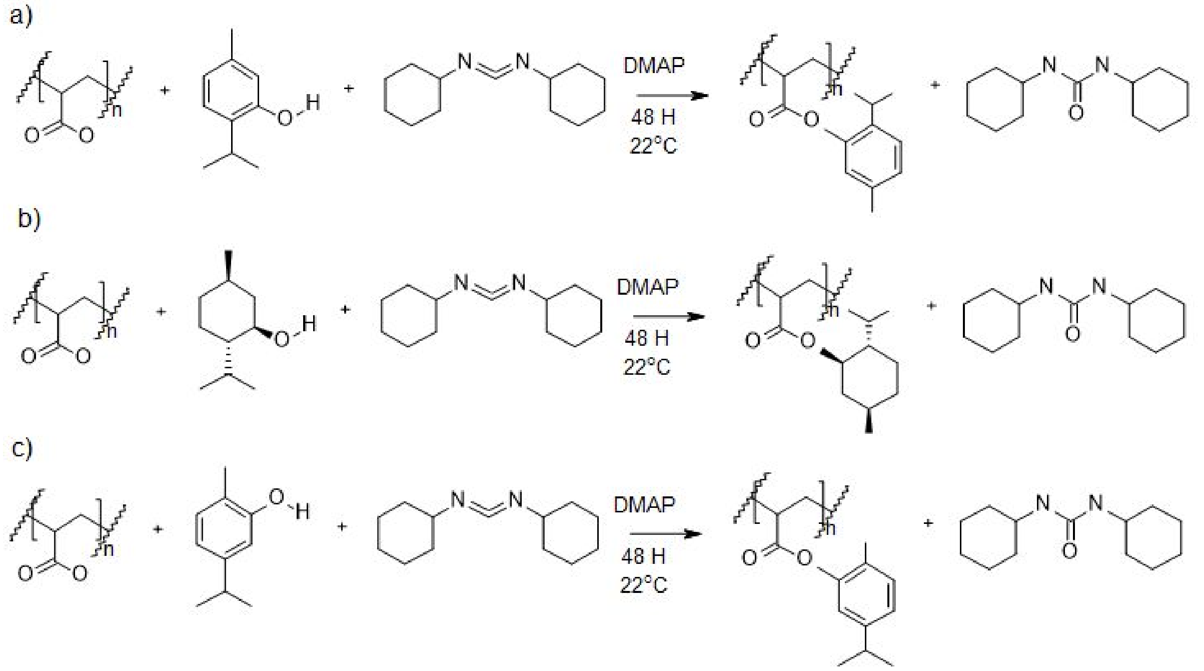

Scheme S2. *Synthesis of PAA functionalised with a) thymol, b) mentol, and c) carvacrol obtained by Steglich esterification.*

Figure 1 shows the changes in light transmission over 72 hours of scanning samples of the designed PESCs with curcumin at a height of 20 mm. Analysis of the transmission curves indicates that self-organization in polyelectrolyte-surfactant systems in the presence of curcumin causes formation of functional nanoscale systems showing different turbidity. In the tested systems, the changes in transmission during 72 h are within narrow range, indicating that the systems are stable. The analysis of the transmission dependencies obtained for CUR/PAA/C12-D_CNMe_3Br shows that the difference between the curves corresponding to the first scan (t=0) and the last one (t=72 h) is approximately 4% (from 74.4 to 78.5%), and the average nanoparticle migration rate V(t) is 0.191 μm/min. The smallest range of changes in *T* was observed for CUR/PAA-MEN-15/C_{12}-D_CNMe_3Br (T 44.7 to 46.9%, V = 0.244 μm/min), while for CUR/PAA-CAA-15/C_{12}-D_CNMe_3Br, T changes from 40 to 45%, at V(t) = 0.168 μm/min. The most turbid suspension and the least stable system is the CUR/PAA-THY-15/C_{12}-D_CNMe_3Br, where the T changes are lower and oscillate between 24.3% to 29%. This system is the least stable and the particles move at the highest velocity V(t)=0.807 μm/min.

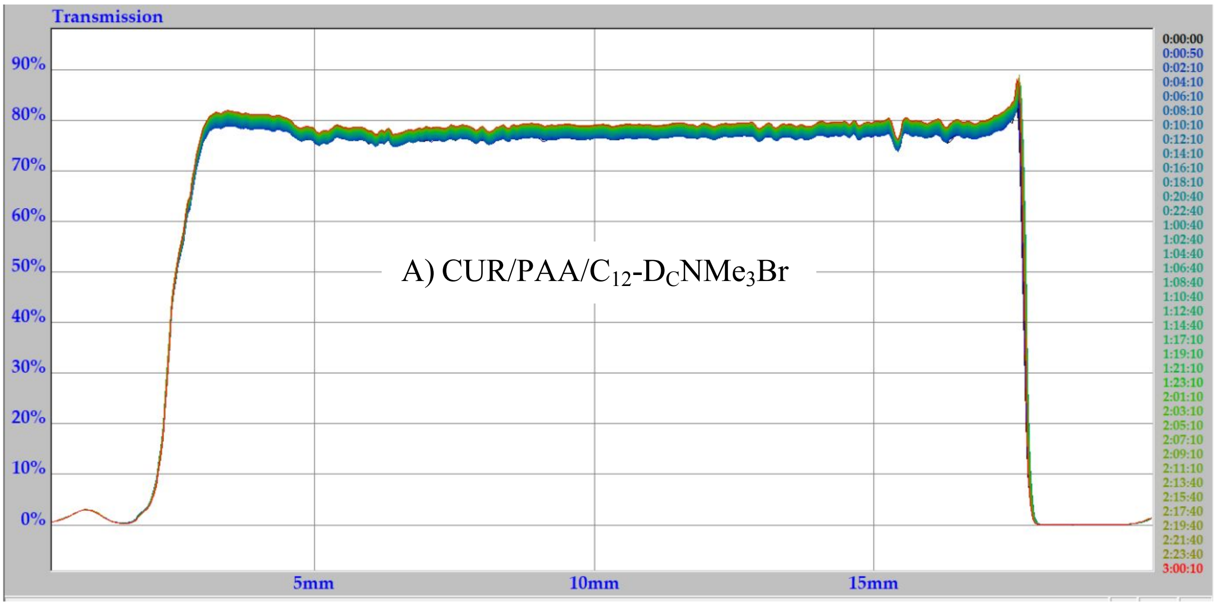
Transmission
90%
80%
70%
60%
50%
40%
30%
20%
10%
0%
5mm
10mm
15mm
A) CUR/PAA/C_{12}-$D_C$$NMe_3$Br
0:00:00
0:00:50
0:02:10
0:04:10
0:06:10
0:08:10
0:10:10
0:12:10
0:14:10
0:16:10
0:18:10
0:20:40
0:22:40
1:00:40
1:02:40
1:04:40
1:06:40
1:08:40
1:10:40
1:12:40
1:14:40
1:17:10
1:19:10
1:21:10
1:23:10
2:01:10
2:03:10
2:05:10
2:07:10
2:09:10
2:11:10
2:13:40
2:15:40
2:17:40
2:19:40
2:21:40
2:23:40
3:00:10

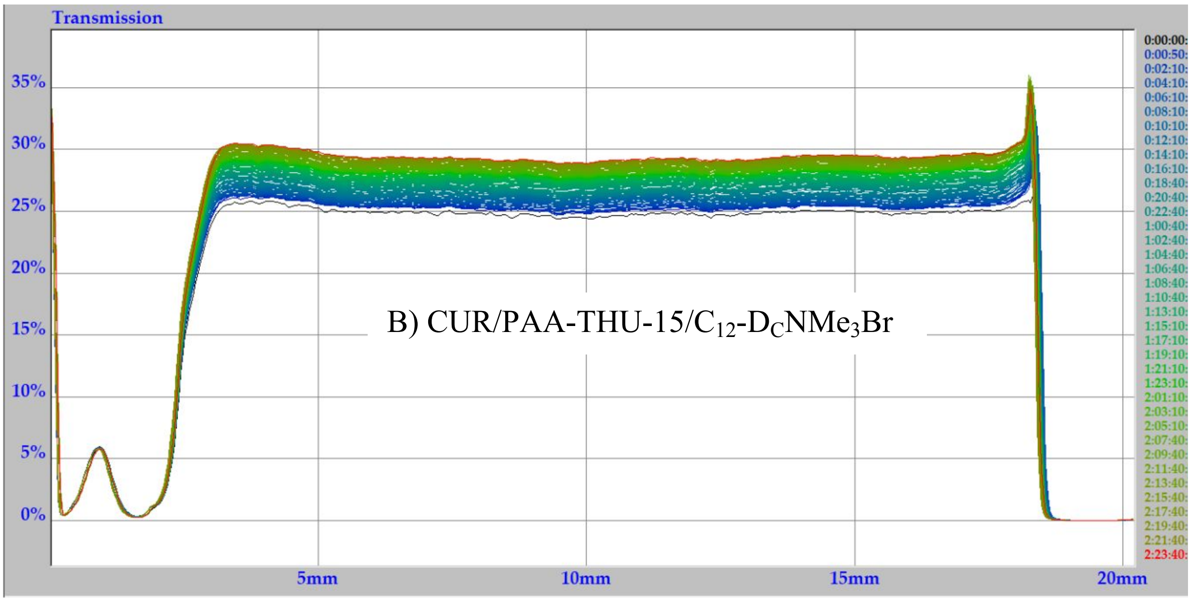
Transmission
35%
30%
25%
20%
15%
10%
5%
0%
5mm
10mm
15mm
20mm
B) CUR/PAA-THU-15/C_{12}-$D_C$$NMe_3$Br
0:00:00:
0:00:50:
0:02:10:
0:04:10:
0:06:10:
0:08:10:
0:10:10:
0:12:10:
0:14:10:
0:16:10:
0:18:40:
0:20:40:
0:22:40:
1:00:40:
1:02:40:
1:04:40:
1:06:40:
1:08:40:
1:10:40:
1:13:10:
1:15:10:
1:17:10:
1:19:10:
1:21:10:
1:23:10:
2:01:10:
2:03:10:
2:05:10:
2:07:40:
2:09:40:
2:11:40:
2:13:40:
2:15:40:
2:17:40:
2:19:40:
2:21:40:
2:23:40:

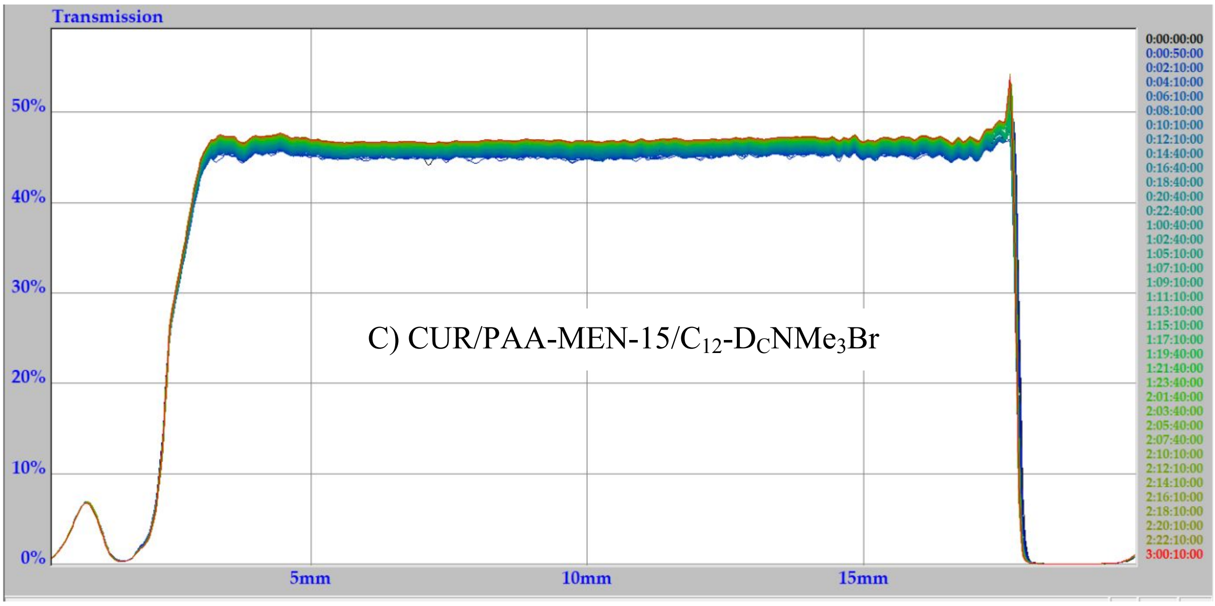

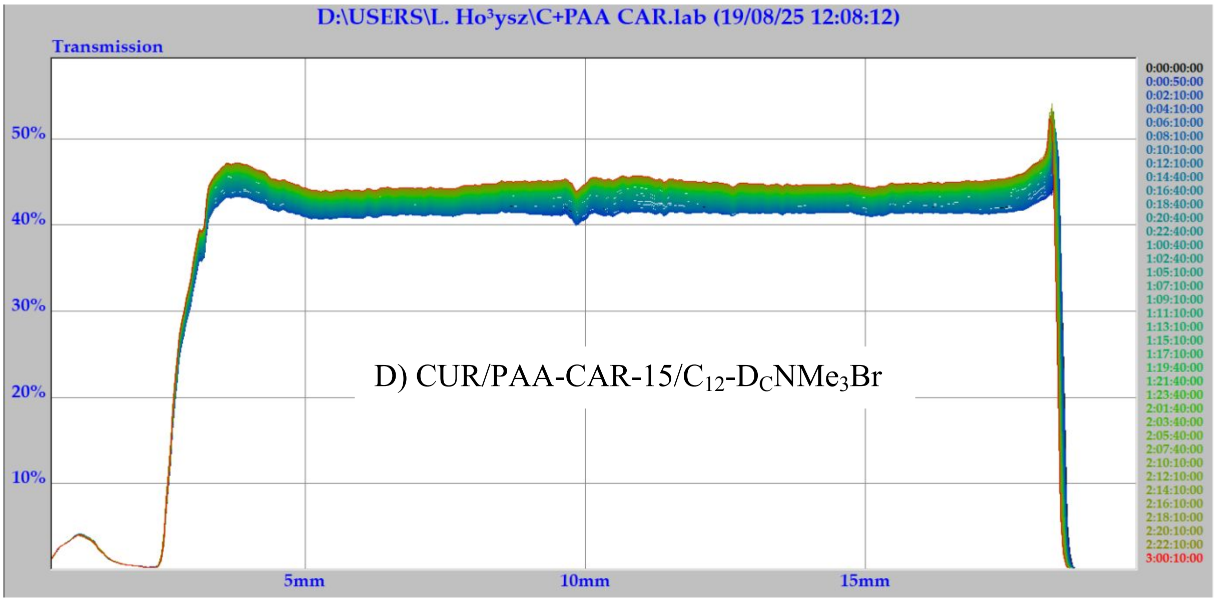

Figure S2. *Turbiscan transmission profiles of the designed PESCs with curcumin A) CUR/PAA/C_{12}-D_CNMe$_3$Br; B) CUR/PAA-THY-15/C_{12}-D_CNMe$_3$Br, C) CUR/PAA-MEN-15/C_{12}-D_CNMe$_3$Br and D) CUR/PAA-CAR-15/C_{12}-D_CNMe$_3$Br.*
